# Abundance of Apple Maggot, *Rhagoletis pomonella*, Across Different Areas in Central Washington, with Special Reference to Black-Fruited Hawthorns

**DOI:** 10.1673/031.012.12401

**Published:** 2012-11-01

**Authors:** Wee L. Yee, Michael W. Klaus, Dong H. Cha, Charles E. Linn, Robert B. Goughnour, Jeffrey L. Feder

**Affiliations:** ^1^ USDA-ARS, Yakima Agricultural Research Laboratory, 5230 Konnowac Pass Road, Wapato, WA 98951; ^2^ Washington State Department of Agriculture, 21 North 1st Avenue, Suite 103, Yakima, WA 98902; ^3^ Department of Entomology, Cornell University, 630 W North Street, Geneva, NY 14456; ^4^ Washington State University, Vancouver, Research and Extension Unit, 1919 NE 78th Street, Vancouver, WA 98665; ^5^ Department of Biological Sciences, 100 Galvin Life Sciences Center, University of Notre Dame, Notre Dame, IN 46556; ^6^ Current address: USDA-ARS, Yakima Agricultural Research Laboratory, 5230 Konnowac Pass Road, Wapato, WA 98951

**Keywords:** *Crataegus douglasii*, Diptera, larval infestation levels, Tephritidae, trapping

## Abstract

The apple maggot fly, *Rhagoletis pomonella* (Walsh) (Diptera: Tephritidae), infests non-commercial apple (*Malus domestica* (Borkh.) Borkh.) and native black-fruited hawthorns (mostly *Crataegus douglasii* Lindl.) in central Washington, but little has been published on the abundance of the fly in this region. In this paper, the abundance of *R. pomonella* across different sites near apple-growing areas in central Washington is documented in order to assess the threat of the fly to commercial apple orchards. The fly was first detected on traps in Klickitat, Yakima, and Kittitas Counties in 1981, 1995, and 1997, respectively. From 1981–2010 in Kittitas and Yakima Counties, only 0 to 4.7% of traps on apple, crabapple, and hawthorn trees were positive for flies, whereas in Klickitat County, located farther from commercial apple orchards, 0 to 41.9% of traps were positive. In 2008, in Yakima County and Goldendale in Klickitat County, 7.8% of black-fruited hawthorn trees were infested, with 0 to 0.00054 larvae per fruit. In 2010, in Kittitas and Yakima Counties and Goldendale in Klickitat County, 25.0% of *C. douglasii* trees were infested, with 0.00042 to 0.00248 larvae per fruit. In 2010, in a remote forested area of Klickitat County far from commercial apple orchards, 94.7% of *C. douglasii* trees were infested, with 0.20813 larvae per fruit. Overall results suggest *R. pomonella* is unlikely to develop high populations rapidly near major commercial apple-growing areas in central Washington, including in black-fruited hawthorns, increasing chances it can be kept out of commercial orchards.

## Introduction

The apple maggot fly, *Rhagoletis pomonella* (Walsh) (Diptera: Tephritidae), is a quarantine pest of apples (*Malus domestica* (Borkh.) Borkh.) in the Pacific Northwest of the U.S. The species evolved on hawthorns (*Crataegus* spp.) in eastern North America (Bush 1966; Bush and Smith 1998) and Mexico (Rull et al. 2006) and moved from hawthorn onto apple about 150 years ago in the eastern U.S.A. (Walsh 1867), differentiating over time into hawthorn and apple host races (Feder et al. 1988; Bush and Smith 1998). The fly apparently was introduced from eastern North America into the West Coast of the U.S.A., perhaps as early as 1951 into Oregon (AliNiazee and Wescott 1986). The first definitive detection of the fly in the West Coast was of larvae infesting backyard apples in 1979 in Portland, Oregon (AliNiazee and Penrose 1981). In 1980, the fly was detected in nearby Vancouver, Washington, and in 1982, in Spokane, Washington; in 1983–1984 it was detected in California and in Utah (Brunner 1987). In western Washington, the fly infests apples and native and introduced hawthorns (Tracewski et al. 1987). Two native black-fruited hawthorns, black hawthorn, *C. douglasii* var. *douglasii* Lindl., and Suksdorf's hawthorn, *C. suksdorfii* (Sarg.) Kruschke, are infested by *R. pomonella* in Washington (Yee and Goughnour 2008).

Central Washington is the center of the largest apple industry in the U.S.A., producing most of the apples in the state of Washington, which were worth $1.4 billion in 2009 (USDA 2010). There is zero tolerance for *R. pomonella*-infested apples (WSDA 2001), making the mere presence of the fly near an orchard a potential threat. However, little work has been published on the abundance of *R. pomonella* in central Washington. As a result, there is a lack of information about the general status of the fly as a threat to commercial apples in the region, information that could be of great value for management efforts in Washington and for allaying concerns of regulatory officials in apple export markets. In central Washington, apples appear less utilized than black-fruited hawthorns (Yee 2008), raising the possibility that hawthorns rather than feral apples in some sites could harbor high fly populations that can threaten apple orchards, but few data are available on fly abundance in these hawthorns.

In this paper, the abundance of *R. pomonella* across different sites near apple-growing areas in central Washington is documented in order to assess the threat of the fly to commercial apple orchards. Fly abundance was determined by fly catches during a long-term detection survey in apples, crabapples, and hawthorns, and by larval infestation levels in *C. douglasii* and *C. suksdorfii*. The implications of the findings for pest management of flies in central Washington are discussed.

## Materials and Methods

### Adult trapping survey in central Washington (1981–2010)

**Figure 1.**
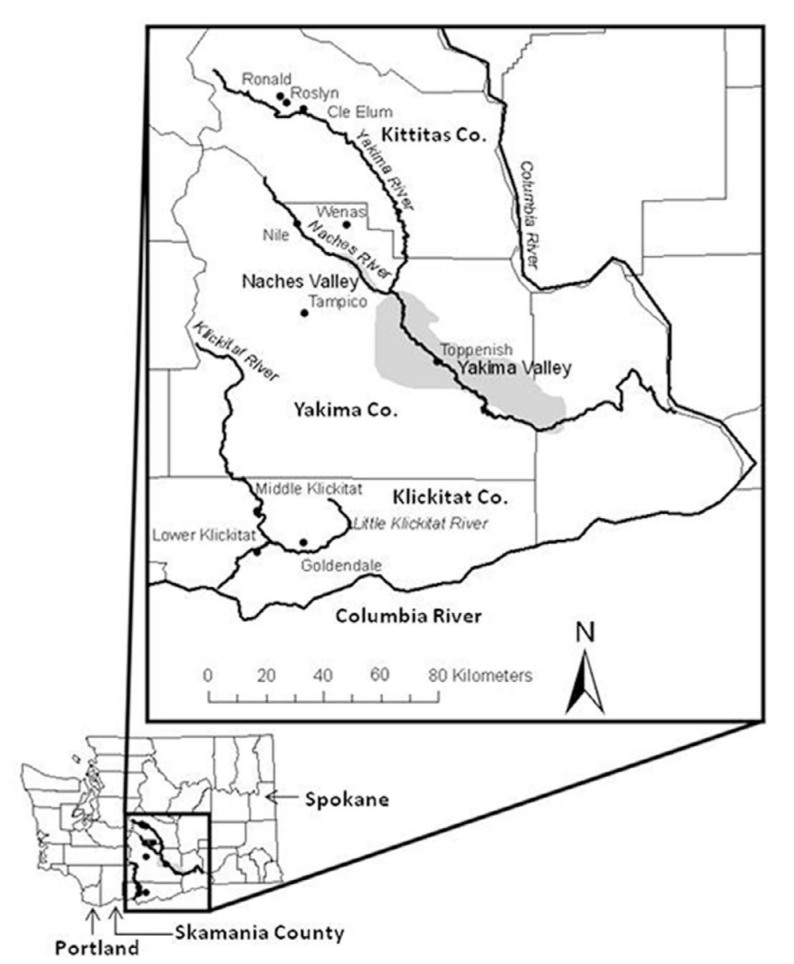
Kittitas, Yakima, and Klickitat Counties in central Washington state where trapping for *Rhagoletis pomonella* was conducted from 1981–2010. County boundaries are indicated by light shaded lines. Also shown are sites where *R. pomonella* abundance in native hawthorns was determined in 2008 and 2010, shown in relation to the Naches and Yakima Valleys (shaded) and major rivers. Cascade Mountain range runs north-south west of area shown in box. High quality figures are available online.

Washington State Department of Agriculture's (WSDA) annual statewide apple maggot detection survey began in 1981, shortly after *R. pomonella* was detected in Portland (Brunner 1987). The goal of the survey since its inception was to delimit the spread of the fly and keep it out of commercial apple orchards. The survey work reported here was conducted in Kittitas, Yakima, and Klickitat Counties (Figure 1) from 1981–2010, essentially spanning the period since *R. pomonella* was first detected in apple in the Portland area until the time of this paper. Most areas in Kittitas, Yakima, and Klickitat Counties (Figure 1) are relatively dry ponderosa pine or bunchgrass and sagebrush habitats (Lyons and Merilees 1995). The ponderosa pine habitats in Kittitas and Klickitat Counties are wetter than the bunchgrass and sagebrush habitat in Yakima County. Major commercial apple orchards are found throughout the Yakima Valley as well as the Naches Valley (gray areas in Figure 1). The survey emphasized trapping in these areas.

**Figure 2.**
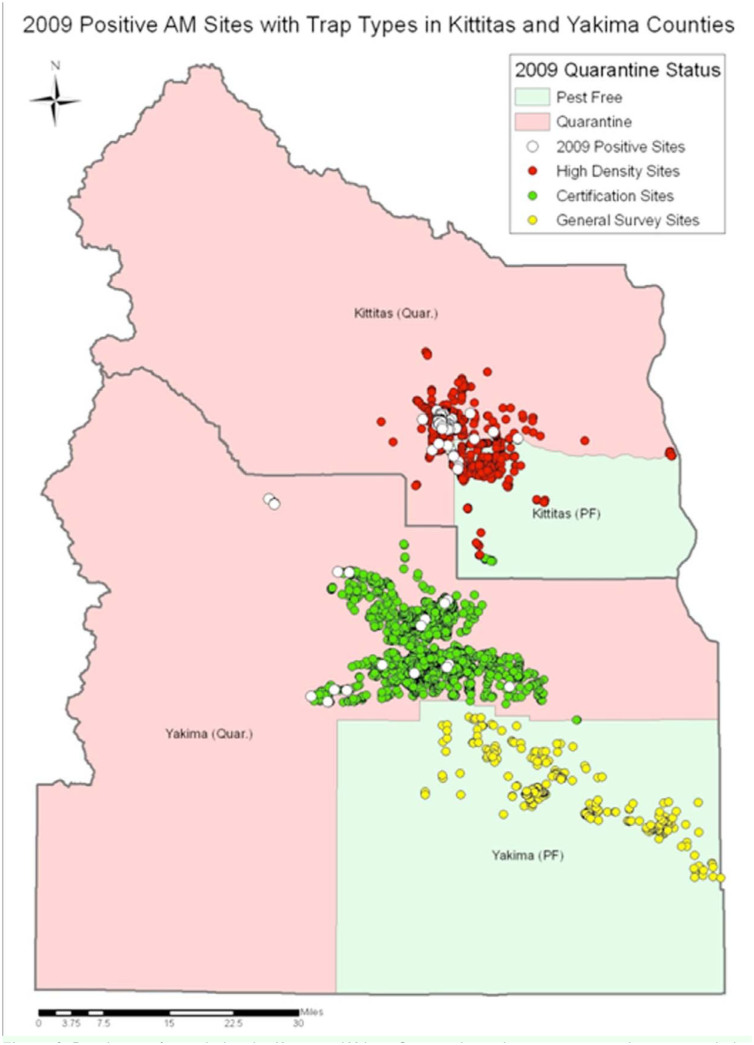
Distribution of traps deployed in Kittitas and Yakima Counties during the trapping survey, showing traps deployed in 2009. AM = apple maggot (*Rhagoletis pomonella*). Totals of 3,044 and 3,853 traps were deployed in Kittitas and Yakima Counties, respectively. High quality figures are available online.

Flies were captured over the course of the 30year monitoring period using yellow sticky traps (Pherocon® AM (apple maggot) traps (Zoecon Co., http://zoecon.com/) or equivalent) baited with one ammonium carbonate or bicarbonate lure containing 10 g of the chemical attractant. Traps were hung in non-commercial apple trees, crabapple, and ornamental hawthorns from mid-June to late September in backyards, roadsides, and rural settings, including wilderness areas. Native hawthorns were trapped less frequently due to a scarcity of these plants in trapped areas. Each tree had one trap that was replaced every 2–4 weeks, as the trap became covered with debris. The survey traps were categorized as three types: (1) general survey traps were placed on host trees at a density of roughly one per 2.6 km^2^ (1 mile^2^) in places where no flies had previously been detected; (2) high-density traps were placed in all accessible host trees 0.8 to 1.6 km (½ to 1 mile) around positive fly trees, and were used to delimit the scope of newly detected fly infestations; (3) certification traps were placed in the 1.9 km (1.2 mile) buffer zone around apple orchards, and were deployed in Kittitas and Yakima Counties (many large commercial orchards present) but not in Klickitat County (few commercial orchards present). Certification trapping is required for the exportation of apples from quarantined areas and may be required for exporting apples from fly-free zones. Traps were deployed in pest-free and quarantine areas. Pest-free areas are those where the larval stage of *R. pomonella* have not been found; quarantine areas are those where adults and larvae have been found. Figure 2 shows the typical distribution of the three trap types deployed in Kittitas and Yakima Counties during the trapping survey (in 2009 in this case). The distribution approximately mirrored that of host-trees present near apple-growing areas in the Yakima Valley; areas immediately outside this distribution were generally non-irrigated sagebrush habitat with few or no trees. Klickitat County had not been trapped in some years. Otherwise, numbers of traps varied from 6 to 4,482 per county over the 30 years of the survey. Many of the same trees were trapped every year of the study. Flies were removed from traps every 1–3 weeks each season. As part of a management program, trees that trapped positive for flies were generally treated weekly with applications of the insecticide Imidan (Gowan Co., http://www.gowanco.com/) by county pest control boards. *R. pomonella* were differentiated from *Rhagoletis zephyria* Snow, the snowberry maggot, based on Wescott (1982). Host-tree species were not always separated in the detection survey, and so trap records from different host trees were combined.

### Larval abundance survey in hawthorns in central Washington (2008 and 2010)

In addition to the trapping studies of adult abundance, larval surveys were conducted at 10 field sites in 2008 and 2010 to confirm *R. pomonella* infestation in hawthorn fruit. Although unlikely, it is possible that flies captured on yellow traps could conceivably have originated not from hawthorn but elsewhere (e.g., from local apple sources), or that fly larvae feeding in hawthorns were not *R. pomonella* but *R. zephyria*. The larval abundance surveys allowed for these alternative scenarios to be rejected and for estimates of local fly densities to be made. The host-plant identified for *R. zephyria* in Washington is snowberry, *Symphoricarpos albus* (L.) Blake (Bush 1966; Yee 2008). Table 1 and Figure 1 show the 10 hawthorn fruit collection sites surveyed for larval abundance. These sites were located within the WSDA adult trapping survey areas, but no records were found showing native hawthorn trees used for collections had been trapped by WSDA, as emphasis had been placed on monitoring feral apples, crabapples, and ornamental hawthorns (Table 1, site trapping history in footnotes). *Crataegus douglasii* and/or *Crataegus suksdorfii* fruit were collected at the 10 sites in 2008 and 2010. Hawthorn identifications were based on Brunsfeld and Johnson (1990). In Ronald, Roslyn, and Cle Elum, and in Wenas, Nile, Tampico, and Toppenish, only *C. douglasii* fruit were collected, although *C. suksdorfii* may have been present but not seen. In Klickitat sites, a survey of local hawthorn trees inidcated that 86% (n = 29 total) were *C. douglasii*, with the remainder *C. suksdorfii*; in Goldendale, 75% were *C. douglasii* (n = 96 total). No native red-fruited hawthorns (Phipps 1998) were found at the 10 collection sites.

In 2008, randomly selected black-fruited hawthorn fruit from trees at Nile (19 trees), Wenas (40 trees), Tampico (21 trees), Toppenish (4 trees), and Goldendale (34 trees) sites were sampled from mid-August to late September. The number of trees sampled at the sites reflected a combination of how many trees were growing in the area that could be accessed and available labor for picking fruit. Fruit on trees among and within sites ripened asynchronously, but all fruit was collected when it was ripe and black. *Crataegus douglasii* and *C. suksdorfii* at the Goldendale site in 2008 were not distinguished and, thus, pooled results are presented for this site in 2008. However, all other samples were distinguished by host-tree species. Collected fruit were placed in 3.8-L zip lock plastic bags, transported back to the laboratory, and held in an -25° C constant temperature room with a 16:8 L:D photoperiod. Each fruit sample was weighed when fresh. From one sample, 40 randomly selected fruits were weighed in order to estimate the number of fruit collected in the sample. Fruit were kept in bags for two to four weeks and then, if necessary, placed in tubs at ∼20% relative humidity to prevent or slow rotting and molding. Bags and tubs were checked for puparia (used synonymously with larvae here) two or three times a week for six weeks. Puparia were held at 20–22° C for around three weeks, stored in moist soil, chilled at 3– 4° C for six months, and then held at 26–27° C and 16:8 L:D for adult emergence. Adult flies that emerged following laboratory rearing were identified (Wescott 1982). Voucher specimens were kept at the insect collection at the USDA-ARS Yakima Agricultural Research Laboratory in Wapato, WA.

In 2010, fruit from randomly selected *C. douglasii* trees at Ronald, Roslyn, and Cle Elum (19 trees from the 3 sites combined), Wenas (18 trees), Middle Klickitat and Lower Klickitat (19 trees), and Goldendale sites (15 trees) (Figure 1, Table 1) were sampled from mid-August to early October. In addition, *C. suksdorfii* trees at Middle and Lower Klickitat (5 trees) and Goldendale sites (9 trees) (Figure 1, Table 1) were sampled from August to September. Nile, Tampico, and Toppenish sites were dropped in 2010 due to labor limitations, as labor was diverted to Roslyn and Klickitat sites in 2010 because those trees had not been previously sampled, and we wanted to document a wider range of sites. Some trees sampled at Wenas and Goldendale had also been sampled in 2008. Fruit were maintained and puparia were handled as described above for the 2008 collections, except that puparia were held at 20–21° C for 10 weeks under an 8–12 hour day length for five days per week, and two days in darkness. These conditions resulted in the emergence of “non-diapausing” adults over the 10-week rearing period, allowing for rapid species confirmations of flies from samples.

### Statistics

Data from the 1981–2010 adult trapping survey and from the host rearing studies were primarily descriptive and were not subjected to statistical analyses. For the fly detection survey, traps were deployed for two to four weeks, but the exact numbers of days were not always recorded, preventing calculations of numbers of flies captured per trap per day. Instead, the measure used to standardize fly abundance across years was percentage of traps (one trap per tree) positive for at least one fly.

## Results

### Adult trapping survey in central Washington (1981–2010)

Among the three counties surveyed, *R. pomonella* was first caught in 1981 in Klickitat County on non-commercial apple, in 1995 in Yakima County at Union Gap (47.00, -120.55), and in 1997 in Kittitas County at Ellensburg (46.55, -120.48) (host affiliations for the latter two records are unclear) (Table 2). After these first recorded catches, one fly was caught in 1982 in the town of Klickitat (number of traps uncertain), about 3 km south of the Lower Klickitat site (Figure 1). In 1984, one fly was caught on 70 traps at Goldendale. In the same year, 12 flies were caught on 58 traps in the town of Klickitat. Over the 30 years in Kittitas and Yakima Counties, only 0–4.7% of the traps on apple, crab apple, and hawthorn trees were positive for flies, whereas in Klickitat County, south of Kittitas and Yakima Counties (Figure 1), 0–42.9% of traps were positive. In general, more flies were detected in Klickitat than in Kittitas County, and more in Kittitas than Yakima County over the 30 years when > 30 traps were deployed. The mean percentages of traps capturing flies (averaged from columns in Table 2) in Kittitas, Yakima, and Klickitat Counties were low: 1.5, 0.6, and 6.5%, respectively, starting when flies were first detected at these sites. In contrast, trap catches at western Washington sites on apple trees were high: 100% of yellow panels captured at least one fly during the same time period as traps in central Washington were deployed in 2003–2005 (Yee et al. 2005). In Klickitat County from 2001–2006 (except 2005), traps were only placed in a few trees in the dry, extreme eastern portion of the county to verify the absence of flies there. As a result, fly catches during this period reflected location differences and trapping intensity rather than reductions in fly abundance in Klickitat County. Fluctuations in fly catches in Kittitas and Yakima Counties among years could be due to emphasis being placed in some years on trapping trees that were positive for flies in previous years (e.g., around Ellensburg in Kittitas County beginning in 2003 and Nile in Yakima County in 2004 and 2005; Table 2). Thus, in some years, overall fly abundance was probably lower than suggested by the survey.

### Larval abundance survey in hawthorn in central Washington in 2008

In 2008, in Yakima County and Goldendale in Klickitat County, a total of 7.8% of black-fruited hawthorn trees were infested, with a range of infestation of 0–0.00054 larvae per fruit among sites (Table 3). Regardless of when and where hawthorns were sampled in central Washington in 2008, population densities were low. As a basis for comparison, average larval infestation levels of *C. douglasii* at one site in Skamania County in western Washington in 1982, 1983, and 1984 were 0.027, 0.060, and 0.015 per fruit, respectively (Tracewski et al. 1987), or 28–111 times greater than the highest infestation rate in 2008 in central Washington.

### Larval abundance survey in hawthorns in central Washington in 2010

In 2010 in Kittitas and Yakima Counties and Goldendale in Klickitat County, a total of 25.0% of *C. douglasii* trees were infested, with a range of infestation of 0.00042–0.00248 larvae per fruit among sites (Table 4). In 2010, in a remote forested area of Klickitat County (Middle and Lower Klickitat sites combined) relatively far from commercial apple orchards (Figure 1), 94.7% of *C. douglasii* trees were infested, with 0.20813 larvae per fruit (Table 4). This represents the one exception to the general observation that infestation levels in black-fruited hawthorns in central Washington are low.

## Discussion

Over a period of 30 years, only 0–4.7% of traps on apple, crabapple, and hawthorn trees in Kittitas and Yakima Counties were positive for *R. pomonella*, implying that human enacted management measures and/or natural factors have prevented fly populations from reaching levels seen in western Washington (Tracewski et al. 1987; Yee et al. 2005; Yee and Goughnour 2008). The *R. pomonella* monitoring and targeted insecticide control program implemented by the WSDA and county pest control boards during this period could have been partly responsible for slowing the fly's spread. However, natural factors probably have played a larger role, because insecticides for fly control were and are not routinely used in the Roslyn area, Wenas, or Nile (sprayed only once in fall 2004), but these sites still have low abundance *of R. pomonella*. Some aspects of the physical environment are likely keeping these populations in check. One possibility is the climate, which is drier and hotter during the summer and fall than the climate west of the Cascade Mountains in Washington. *R. pomonella* also occurs in higher abundance in hawthorns in western New York (Reissig and Smith 1978), where it is relatively wet, than in central Washington. The low fly populations near or in apple-growing regions in central Washington is probably one reason why no exported commercial apples from this region have been found to be infested with *R. pomonella* larvae (WSDA 2010), even though feral apples in central Washington are occasionally infested (Yee 2008).

Data from the 30-year detection survey suggest that *R. pomonella* was slow to spread and establish near or within the apple-growing regions in Kittitas and Yakima Counties. The trapping survey first detected the fly in Klickitat County 13 and 15 years before it was found in Kittitas and Yakima Counties, respectively. These data suggest that the fly may have spread relatively rapidly to Klickitat County following its introduction to the Pacific Northwest, but has been slower in expanding its range eastward from Klickitat, perhaps due in part to the difficulty of dispersal from forested to more barren sagebrush habitats with isolated trees and/or reduced opportunity for human mediated transport of infested fruit, likely via apple. The fly could have expanded its range faster than the data indicate if the fewer traps deployed in Kittitas and Yakima Counties in earlier than later years of the survey (Table 2) were insufficient to detect the low populations. However, this still would not contradict the idea of slow fly dispersal and establishment in Yakima County, because traps that numbered in the hundreds in the earlier years in that county consistently failed to catch a fly. The likelihood of flies being present but not detected would have been greater in Kittitas County from 1988–1996, when 39–123 traps were deployed, than in Yakima County. Data from the 2008 and 2010 larval abundance surveys concur with the trapping survey in indicating much higher abundances of flies infesting *C. douglasii* in Klickitat than in Kittitas and Yakima Counties, thus suggesting slow establishment.

It is not clear why black-fruited hawthorns growing in the remote, forested Klickitat sites seem to be better hosts than ones growing in sites in Kittitas and Yakima Counties. The higher larval infestation levels in *C. douglasii* in Klickitat than in other sites could be due to regional variations in hawthorn fruit quality, resulting in differential larval survival. They could also be due to habitat effects, or, as mentioned before, in regards to general fly populations, an establishment time effect influenced by human transport.

Despite the generally low abundance of *R. pomonella* in black-fruited hawthorns, it is still prudent to diligently monitor hawthorns that are near orchards, because of the zero tolerance for larval infestations and the fact that the hawthorn race of the fly can colonize apples (at least in the eastern U.S.A.) (Reissig and Smith 1978). *R. pomonella* has been reported to travel 666 meters within two weeks (Bourne et al. 1934), so perhaps hawthorns > 666 meters from orchards, especially if separated from them by barren landscape, need only be monitored periodically to ensure populations do not dramatically increase. Flies at the Klickitat sites probably pose little threat to major commercial apple orchards, due to the distance and the geographic barriers (Simcoe Mountains and Satus Pass) between the sites and orchards.

In summary, overall results suggest *R. pomonella* is unlikely to develop high populations rapidly near major commercial apple-growing areas in central Washington, including in black-fruited hawthorns, increasing chances it can be kept out of commercial orchards. Whether the detection survey and insecticide spray program described here have contributed to low fly populations is unclear. Habitat, climate, and genetic factors could contribute to low overall fly populations, a hypothesis that may be experimentally tested.

**Table 1.**
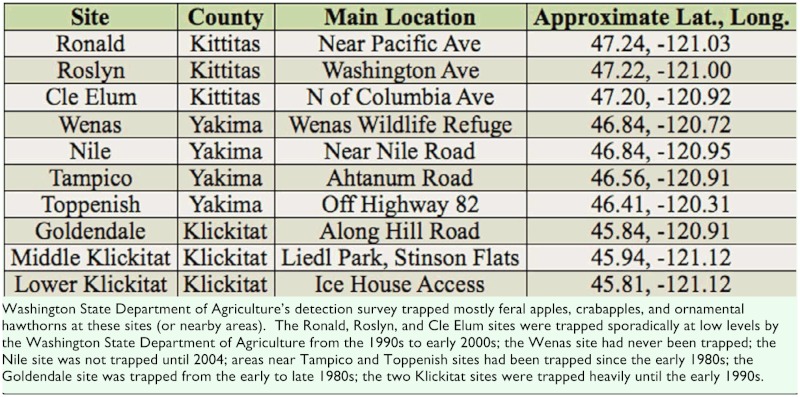
Kittitas, Yakima, and Klickitat County sites sampled for black-fruited hawthorns in 2008 and 2010.

**Table 2.**
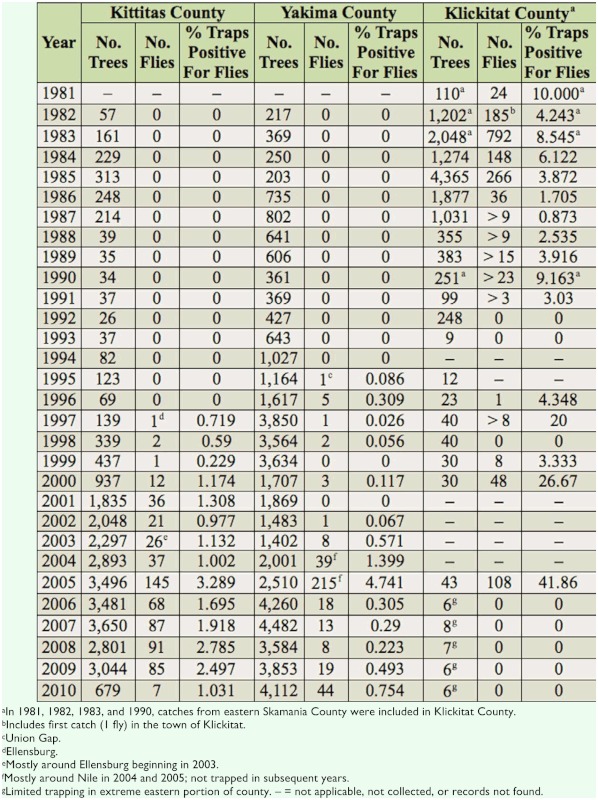
Surveys of *Rhagoletis pomonella* catches on sticky yellow rectangles baited with ammonium carbonate or bicarbonate in native hawthorns, ornamental hawthorn, crabapple, and apple trees in Kittitas, Yakima, and Klickitat Counties in central Washington from 1981–2010. Each tree had one trap at a time over the season, but the trap was replaced when needed. Some trees had multiple fly catches.

**Table 3.**
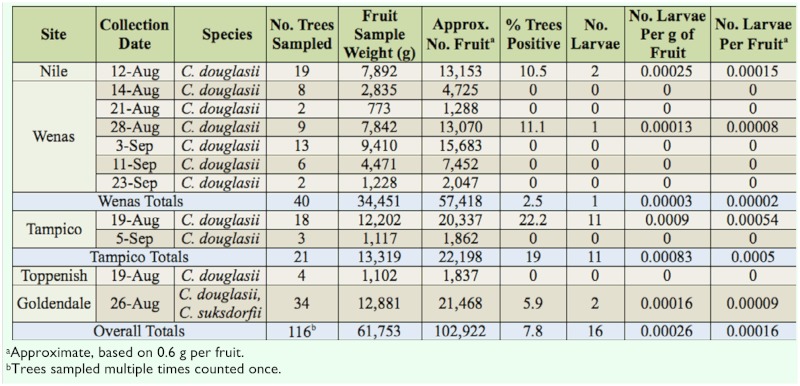
Larval abundance of *Rhagoletis pomonella* in native black-fruited hawthorns in central Washington in 2008.

**Table 4.**
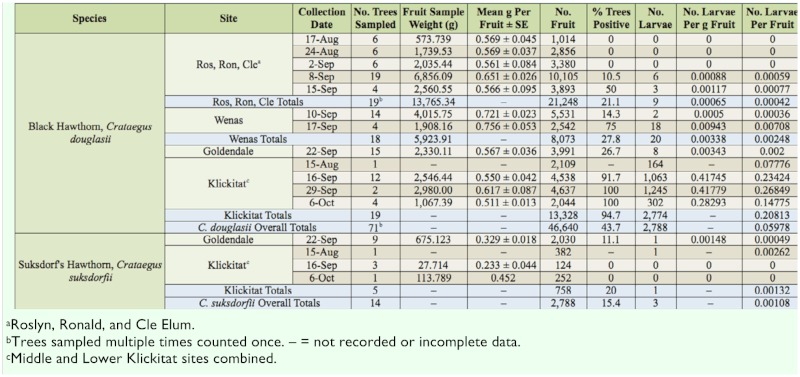
Larval abundance of *Rhagoletis pomonella* in native black-fruited hawthorns in central Washington in 2010.
